# Mechanistic insights into volatile odor changes in surimi gels with typical cross-linking degrees during frozen storage based on lipidomics analysis

**DOI:** 10.1016/j.fochx.2025.102630

**Published:** 2025-06-04

**Authors:** Xiaoying Luo, Guoyan Ren, Shanbai Xiong, Yueqi An, Kang Huang, Yang Hu

**Affiliations:** a College of Food and Bioengineering, Henan University of Science and Technology, Luoyang, Henan 471023, China; b College of Food Science and Technology, Huazhong Agricultural University, Wuhan, Hubei 430070, China; c College of Health Science and Engineering, Hubei University, Wuhan, Hubei 430062, China; d Department of Biological Systems Engineering Washington State University Pullman, WA 99164, USA

**Keywords:** Surimi gel, Frozen storage, Volatile odor compounds, Lipidomics

## Abstract

This study investigated the potential mechanisms underlying odor changes in surimi gel during frozen storage, focusing on lipid alterations. Frozen storage significantly impacted the odor characteristics of surimi gel, with a noticeable decline in fish aroma and an intensification of fishy, oily, and earthy odors after 90 days of freezing. Using liquid nitrogen spray freezing at −80 °C and increasing the cross-linking degree to 62.99 % was beneficial in maintaining the original odor profile of surimi gel. Odor changes during storage were driven by enhanced lipid oxidation and altered lipid distribution. Most lipids significantly associated with odor compounds were unsaturated fatty acids primarily found in phosphatidylcholine (PC) and phosphatidylethanolamine (PE). Notably, PC(14:0_20:5), PC(16:0_20:4)(rep), PC(16:0_20:5), PC(18:0_20:4), PC(18:3e_22:6), PC(20:3_22:6), PE(20:4_20:4), and PE(20:4_22:6)(rep) showed negative correlations with volatile compounds such as acetoin, hexanal, heptanal, benzaldehyde, nonanal, 1-octen-3-ol, and 1-hexanol. The oxidation of these lipids resulted in alterations to the odor profile of the surimi gel.

## Highlights

• Frozen surimi gels' fish aroma decreased, while fishy and greasy odors increased.
• Fishy odor increased in late frozen storage was strongly linked to fat oxidation.
• Oxidative of unsaturated fatty acids in PC and PE caused odor alters in gels.
• Odor compounds notably correlate with unsaturated fatty acids in phospholipids.

## Graphical abstract

**Figure:**
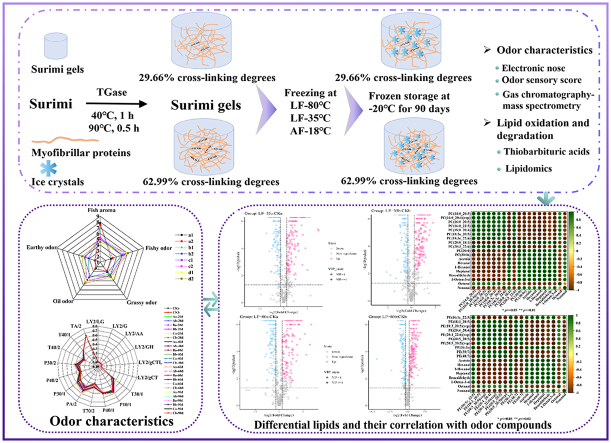

## 1 Introduction

Surimi products are popular among consumers worldwide due to their high nutritional value and unique flavor (An et al., 2024). Flavor is a key factor in the overall acceptability of surimi products. Precursor chemicals undergo several intricate interactions to generate the flavor of aquatic products (An et al., 2022). Volatile compounds produced during frozen storage due to lipid oxidation and degradation have a substantial influence on the formation of flavor in surimi gels. Frozen storage is a crucial method for preserving surimi and its products by inhibiting microbial growth and endogenous enzyme activity (Zhu et al., 2024; Zhu et al., 2025). However, even while frozen storage can successfully postpone chemical processes, it is unable to completely stop fish tissues from undergoing lipid oxidation and hydrolysis, especially when exposed to free radicals, oxygen, pro-oxidant metal ions, and reactive aldehydes. Lipids remain involved in various chemical reactions at low temperatures, thereby impacting the texture, color, odor, and taste of the product (Ying et al., 2025). Key odor compounds in surimi gels, such as hexanal, octanal, nonanal, hexanol, and 1-octen-3-ol, have been identified (Luo et al., 2022; Luo, Huang, Yu, et al., 2024), with 1-octen-3-ol, derived from linoleic acid, imparting a mushroom and metallic odor, caused by lipid degradation and oxidation (Wang, Yu, et al., 2024). Due to the cleavage of C—O bonds in alkoxyl radicals, 9-hydroperoxides from linoleic acid and 9-, 10-, and 11-hydroperoxides from oleic acid can produce hexanal, pentanal, octanal, and heptanal, respectively (Shahidi & Hossain, 2022). Additionally, some flavor compounds are lipid-soluble, and alterations in the lipid composition can affect their concentration (Zhou et al., 2024). Therefore, an in-depth understanding of lipid composition and its reaction pathways is crucial to unravel the mechanisms of flavor quality changes.

Currently, lipidomics has been widely used to quantify all lipids in food systematically. For example, Chen et al. (2023) investigated the effect of freezing treatment on the lipidomics of the large yellow croaker. They found that autophagic animal metabolism and glycerophospholipid metabolism are two significant pathways for lipid changes. Wang, Huang, et al. (2024) examined the mechanisms of salt in the flavor formation of lightly salted large yellow croaker, discovering that salt enhances the activity of lipase, promoting the hydrolysis and oxidation of phospholipids to generate key flavor compounds. Fresh fish possesses a pleasant aroma. However, freezing treatment can induce changes in the composition and concentration of volatile compounds, resulting in the depletion of unique seafood aromas and the deterioration of flavor (Mramba & Mkude, 2022). Previous studies in this project revealed that the fishy odor in surimi gels increased after frozen storage, suggesting a possible correlation with intensified lipid oxidation due to freezing. However, it remains unclear which specific lipids and through which pathways produce volatile compounds detrimental to product flavor quality. Therefore, elucidating the lipid changes in frozen surimi gels lipid changes is important for explaining flavor changes.

Based on this, the present study examined the alterations in odor characteristics of typically cross-linked surimi gels (29.66 % and 62.99 %) during frozen storage at −20 °C by utilizing an odor analyzer, gas chromatography–mass spectrometry (GC–MS) combined with organoleptic evaluation. Lipid composition and changes were further investigated using lipidomics to reveal the potential molecular mechanisms underlying variations in important volatile flavor compounds in typical cross-linked surimi gels treated with different freezing methods during storage at −20 °C from the perspectives of lipid oxidation and degradation. This research will provide valuable insights into the underlying mechanisms by which lipid changes influence odor formation in surimi products during frozen storage, offering crucial theoretical significance and practical value for improving the flavor stability and processing quality of frozen surimi products.

## 2 Materials and methods

### 2.1 Materials and chemicals

Jingli Aquatic Product Co., Ltd. supplied *Hypophthalmichthys molitrix* surimi (AAA grade, Hubei, China). Jin Nuo Gu, Co., Ltd. (Jiangsu, China) provided the transglutaminase (TGase) (3000 U/g). Hexanal, 1-hexanol, heptanal, benzaldehyde, 1-octen-3-ol, octanal, nonanal, acetoin standard reagents, and cyclohexanone were acquired from Sigma-Aldrich Corporation, USA. Methanol and acetonitrile were purchased from Thermo Fisher Scientific, USA.

### 2.2 Preparation of surimi gels with different cross-linking degrees

The preparation of surimi gels followed the procedure of Luo, Huang, Lei, et al. (2024). After thawing frozen silver carp surimi at 4 °C, it was divided into 200 g portions and pre-chopped for 1 min. Subsequently, different concentrations of transglutaminase (0 and 10 U/g) and 2.5 % (*w*/w) NaCl were added, followed by an additional 1 min of chopping. The mixtures were then vacuum-packed into plastic casings, sealed at both ends, and subjected to a two-step heating process (40 °C for 1 h, then 90 °C for 0.5 h) to prepare surimi gels with cross-linking degrees of 29.66 % and 62.99 %, respectively.

### 2.3 Frozen storage treatment of surimi gels under various treatment conditions

The frozen storage treatment of surimi gels was conducted according to the method described by Luo et al. (2025). The surimi gels were split up into four groups at random, with one group serving as the control group (CK). The remaining three groups were frozen in the YDZ-500 liquid nitrogen cabinet freezer (Chengdu Kelais Low-Temperature Equipment Co., Ltd., China) at −35 °C (LF-35 °C) and − 80 °C (LF-80 °C), and in a freezer set at −18 °C (AF-18 °C), respectively. The freezing process was completed when the center temperature of the surimi gels fell below −18 °C.

### 2.4 Odor sensory

Ten pre-trained panelists were selected to conduct a sensory evaluation using five sensory descriptors: fishy aroma, grassy odor, fish odor, earthy odor, and oil odor. A 9-point hedonic scale was employed for the assessment, with scoring criteria based on the method previously reported by Luo, Huang, Yu, et al. (2024).

### 2.5 Overall odor analysis

An electronic nose (FOX4000, Alpha M.O·S, France) was used to determine the alterations in the overall odor profile of surimi gels from different treatment groups during frozen storage. The measurement procedure was based on the method previously outlined by Luo, Huang, Yu, et al. (2024).

### 2.6 Determination of volatile odor compounds

#### 2.6.1 Extraction of volatile odor compounds from surimi gel

Volatile odor compounds from frozen surimi gel were extracted using the direct solvent extraction (DSE) method with dichloromethane, followed by purification through solvent-assisted flavor evaporation (SAFE). The extract was dehydrated, concentrated using a Weiss distillation setup, and then reduced to 1 mL under nitrogen to obtain the concentrated aroma extract (An et al., 2022).

#### 2.6.2 GC–MS analysis of volatile odor compounds

The temperature of the GC injection port was set to 230 °C, and the analysis was performed in splitless mode, using helium as the carrier gas at a flow rate of 1 mL/min. The study was conducted on an HP-5 chromatographic column (30 m × 0.25 mm × 0.25 μm). The temperature was maintained at 40 °C for 2 min and then increased to 250 °C at 4 °C/min for 5 min, and the mass spectrometry was performed in full scan mode (Li, Wen, Xiong, Xiao, & An, 2023). The standard curves for measuring significant volatile odor compounds are presented in Table S1, and their chromatographic profiles are displayed in Fig. S1.

### 2.7 Lipid oxidation measurement

Lipid oxidation was determined based on the method previously reported by Luo, Huang, Yu, et al. (2024).

### 2.8 Determination of lipidomics

#### 2.8.1 Lipid extraction

A total of 25 mg of surimi gels was weighed and placed in a 2 mL thick-walled centrifuge tube, along with two small steel beads. Subsequently, 800 μL of pre-cooled dichloromethane/methanol (3:1, V/V) precipitant was added. The mixture was then ground for 5 min, subjected to ultrasonic treatment in an ice bath for 10 min, and left to stand overnight at −20 °C. The mixture was then centrifuged at 25000*g* for 15 min at 4 °C. The supernatant (600 μL) was transferred to a freeze-dryer for solvent removal and then reconstituted with 600 μL of lipid reconstitution solution (isopropanol: acetonitrile: water = 2:1:1). After shaking for 10 min and undergoing ultrasonic treatment in an ice bath for another 10 min, it was centrifuged again at 25000*g* for 15 min at 4 °C.

#### 2.8.2 Ultra-high-performance liquid chromatography-mass spectrometry analysis

The obtained extract was analyzed using a Waters ultra-performance liquid chromatography (UPLC) I-Class Plus coupled with a Q Exactive high-resolution mass spectrometer for compound separation and detection. The chromatographic conditions employed a CSH C18 column (1.7 μm, 2.1 × 100 mm, Waters, USA). In the positive ion mode, solution A, containing 60 % acetonitrile in water, 10 mmol/L ammonium formate, and 0.1 % formic acid, and solution B, which included 90 % isopropanol, 10 % acetonitrile, 10 mmol/L ammonium formate, and 0.1 % formic acid. The mobile phase in negative ion mode consisted of 60 % acetonitrile in water with 10 mmol/L ammonium formate (solvent A) and 90 % isopropanol with 10 % acetonitrile and 10 mmol/L ammonium formate (solvent B).

The elution gradient was programmed as follows: the proportion of solution B started at 40 % and gradually increased to 43 % within the first 2 min. From 2 to 2.1 min, it rose to 50 %, followed by an increase to 54 % between 2.1 and 7 min. The concentration then spiked from 54 % to 70 % between 7 and 7.1 min, reaching 99 % from 7.1 to 13 min. Afterward, the concentration was reduced from 99 % to 40 % between 13 and 13.1 min, and finally, it remained steady at 40 % from 13.1 to 15 min. The injection volume was 5 μL, the column temperature was 55 °C, and the flow rate was 0.4 mL/min. Mass spectrometric conditions included acquiring both MS1 and MS2 data. The mass scanning range was set from 200 to 2000, with a primary resolution of 70,000 and a maximum injection time of 100 ms. Based on precursor ion intensity, the Top 3 ions were selected for fragmentation, and MS/MS data were acquired. The MS/MS resolution was set to 17,500, and the AGC target was 1e5. The maximum injection time (IT) was 50 ms. The stepped collision energy (stepped nce) was set to 15, 30, and 45 eV. The electrospray ionization (ESI) source parameters were configured as follows: sheath gas flow rate was 40, auxiliary gas flow rate was 10, spray voltage was 3.80 kV in positive ion mode and 3.20 kV in negative ion mode, capillary temperature was set to 320 °C, and the auxiliary gas heater temperature was 350 °C. The typical base peak chromatograms of the sample in both positive and negative ion modes are shown in Fig. S2.

#### 2.8.3 Lipidomics data processing

LipidSearch v.4.1 (Thermo Fisher Scientific, USA) was used to analyze the raw mass spectrometry data, producing a data matrix with the results of lipid molecular identification and quantitative information. LipidSearch's output results were loaded into metaX for preprocessing and further analysis. Lipid molecules having more than 50 % and more than 80 % missing values in the experimental samples were eliminated as part of the data-cleaning process. Missing values were filled using the KNN algorithm (k-nearest neighbor). The data was normalized, and relative peak areas were obtained using the probabilistic quotient normalization (PQN) approach. QC-RLSC (Quality control-based robust loess signal correction) was used to adjust for batch effects. Lipid molecules with a coefficient of variation (CV) higher than 30 % in relative peak areas across all samples were removed.

### 2.9 Data processing and analysis

Each experiment was conducted three times, with the lipidomics data collected in sextuplicate. Results are presented as mean ± standard deviation. Statistical analysis for significance differences was conducted using SAS (ANOVA), with *p* < 0.05 indicating significant differences. Pearson correlation coefficient analysis and graphical representations were performed using Origin 2021 software.

## 3 Results and discussion

### 3.1 Variations in the odor perception of surimi gels across different treatment groups during frozen storage

**Fig. 1:**
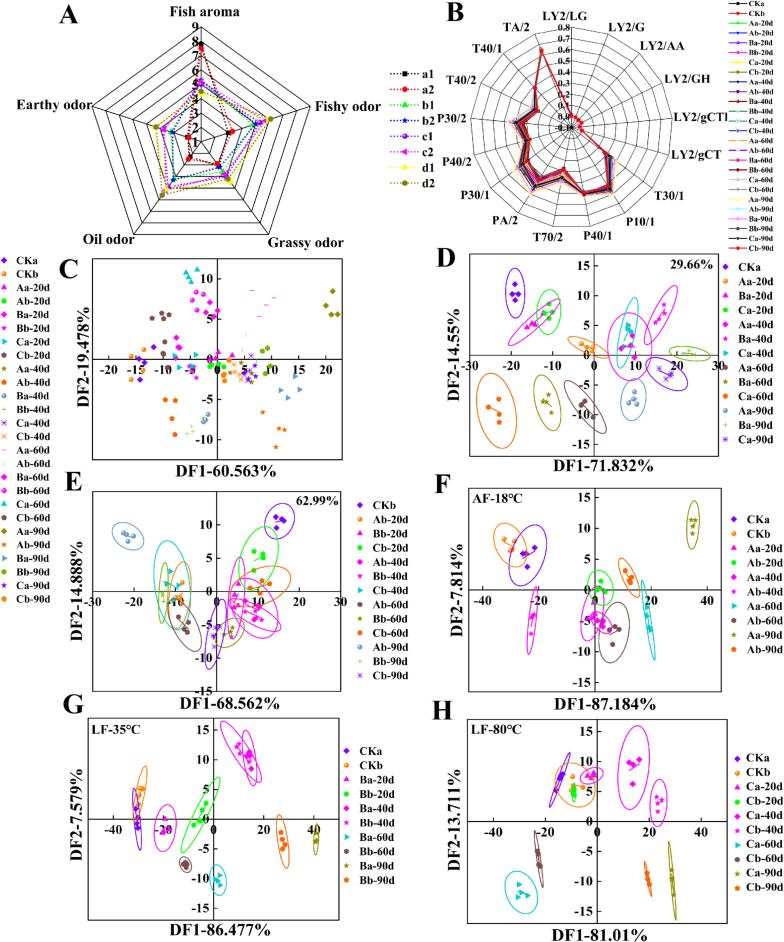
Analysis of odor sensory (A) and odor profile (B—H) during frozen storage of surimi gels in different treatment groups. The letters a, b, c, and d in A represented CK, and surimi gels were frozen at LF-80 °C, LF-35 °C, and AF-18 °C freezing methods and frozen for 90 days, respectively, and 1 and 2 represented surimi gels with 29.66 % and 62.99 % cross-linking degrees, respectively. The uppercase letters A, B, and C in figs. B—H indicated AF-18 °C freezing, LF-35 °C freezing, and LF-80 °C freezing, respectively; The lowercase letters a and b represented surimi gels with 29.66 % and 62.99 % cross-linking degrees, respectively.

The sensory score for the fish aroma in the fresh control group was the highest, dominating the overall odor profile (Fig. 1A). After freezing at AF-18 °C, LF-35 °C, and LF-80 °C and subsequently stored at −20 °C for 90 days, the fish aroma in the surimi gels significantly decreased (*p* < 0.05), with reductions of 44.87 %, 35.90 %, and 33.33 % for the 29.66 % cross-linking degree, and 40.00 %, 30.67 %, and 29.33 % for the 62.99 % cross-linking degree, respectively, compared to the fresh control group. These results indicated that increasing the cross-linking degree of surimi gels reduced odor degradation during storage. After 90 days of storage, the fishy and grassy odors of the surimi gels in the different treatment groups increased significantly (*p* < 0.05) and became the dominant odors of the surimi gels at this time. Additionally, a noticeable increase in grassy and earthy odors was also observed after storage, with the highest odor intensity recorded in the AF-18 °C group. This could be related to the high concentrations of 1-octen-3-ol, hexanal, and heptanal (Guo et al., 2019). Compared to the AF-18 °C group, the LF-80 °C group exhibited better odor sensory scores after storage, retaining the fish aroma more effectively and showing lower intensities of off-odors such as fishy and oil odors.

### 3.2 Alterations in the odor profiles of surimi gels among various treatment groups during frozen storage

As illustrated in Fig. 1B–E, the cumulative discrimination indices of DF1 and DF2 for gels with cross-linking degrees of 29.66 % and 62.99 % remained above 80 % throughout 90 days of storage at −20 °C, indicating that dependent failure analysis (DFA) effectively differentiated odor profiles among freezing treatments. For surimi gels with low cross-linking (29.66 %), odor profiles gradually deviated from the fresh control with prolonged storage, indicating greater odor deterioration. However, the LF-80 °C group remained closer to the fresh sample, suggesting better odor retention. At a higher cross-linking degree (62.99 %), except for AF-18 °C, the odor profiles of other freezing treatments clustered closely, indicating that increased cross-linking effectively preserved the original odor characteristics.

As shown in Fig. 1F–G, the cumulative discrimination indices of DF1 and DF2 exceeded 90 % for surimi gels with varying cross-linking degrees under AF-18 °C, LF-35 °C, and LF-80 °C, indicating that DFA effectively distinguished odor profiles across different cross-linking degrees under the same freezing condition. With extended storage, the odor response regions progressively deviated from those of the fresh control group. In contrast, the LF-80 °C group exhibited more compact clustering and partial overlap with the fresh group at 20 days, indicating better odor retention during early storage. This may be attributed to differences in ice crystal size and structural damage caused by recrystallization.

### 3.3 Quantitative analysis of odor compounds in surimi gels from various treatment groups

**Fig. 2:**
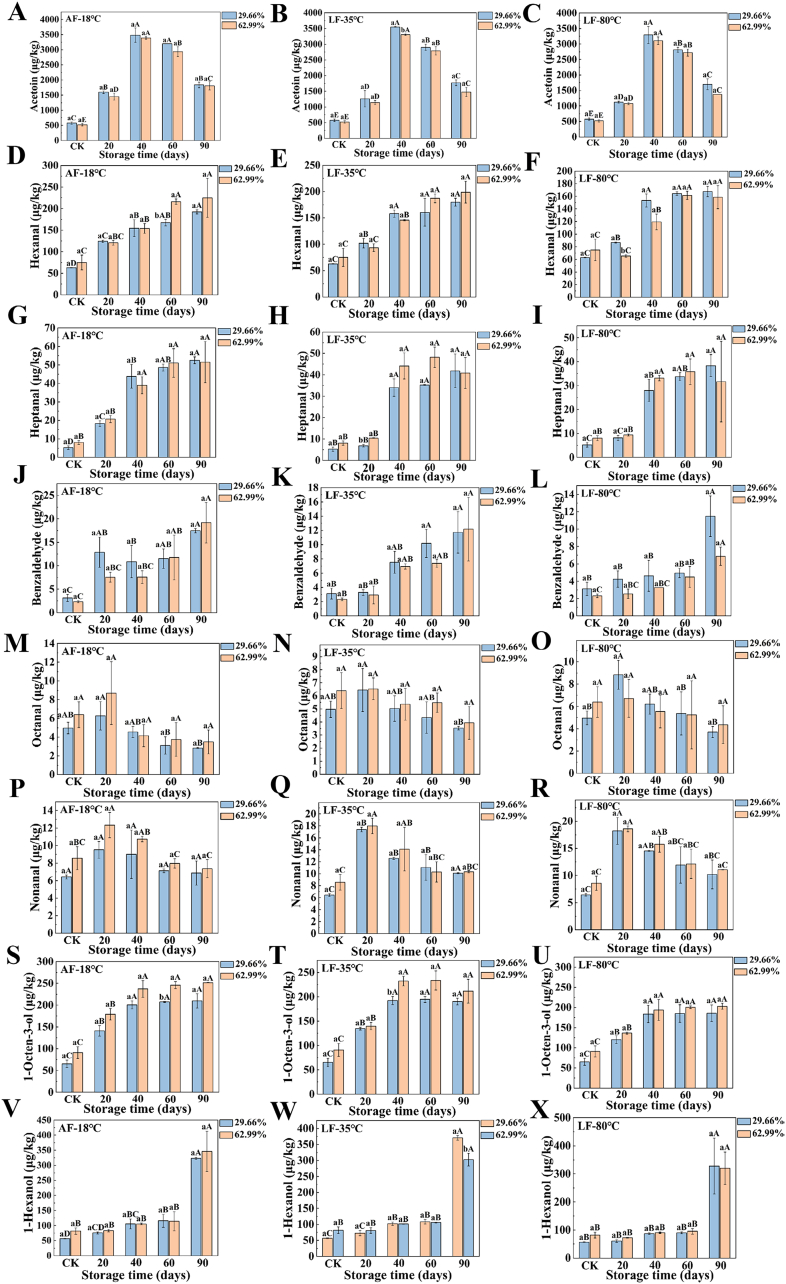
Quantitative analysis of volatile odor compounds in surimi gels with different cross-linking degrees subjected to various freezing methods during frozen storage. Different uppercase letters indicated significant differences in odor compounds in surimi gels under different frozen storage times (*p* < 0.05), and different lowercase letters indicated significant differences in odor compounds in surimi gels with different cross-linking degrees (*p* < 0.05).

The key volatile compounds that have a significant impact on the odor of frozen surimi gels were chosen based on the variable important in the projection (VIP) > 1 in surimi gels with varying cross-linking degrees under different freezing methods obtained from the previous experiments (Luo, Huang, Yu, et al., 2024). These compounds were quantitatively analyzed and included 1-octen-3-ol, 1-hexanol, hexanal, heptanal, benzaldehyde, octanal, nonanal, and acetoin. The quantitative results of volatile odor compounds in surimi gels stored at −20 °C for 90 days are presented in Fig. 2. The content of acetoin in surimi gels from different treatment groups decreased as the cross-linking degree increased and exhibited a pattern of initially rising followed by declining with extended storage times, reaching a maximum at 40 days (Fig. 2A-C).

**Fig. 3:**
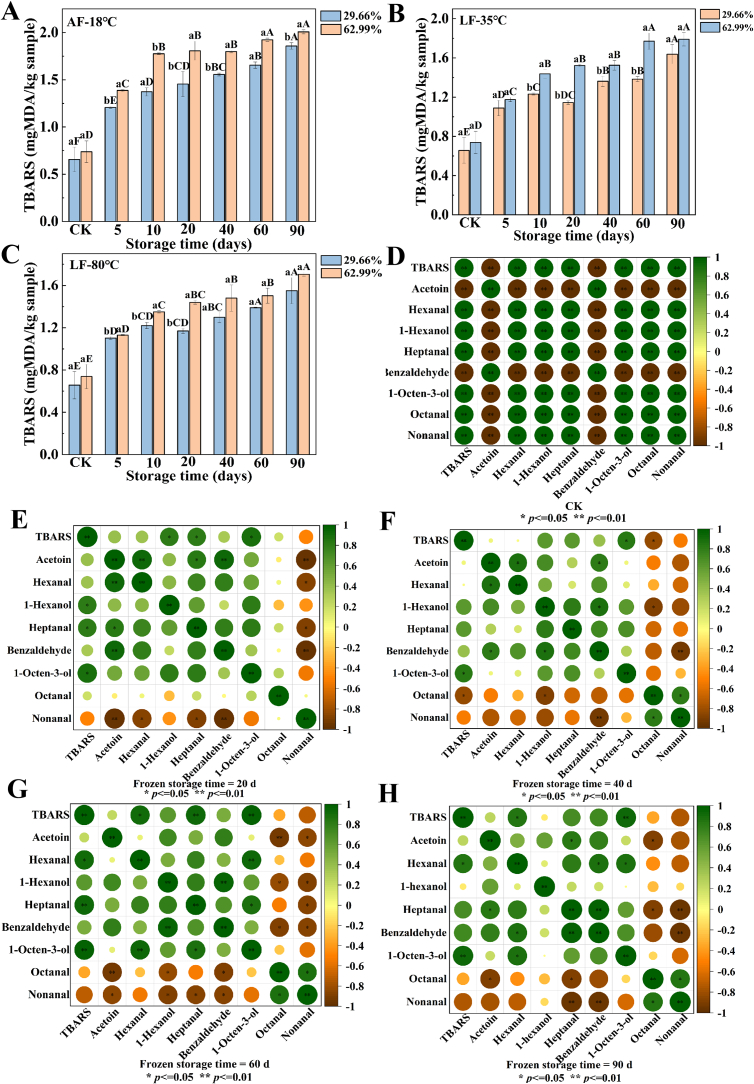
Analysis of lipid oxidation during frozen storage of surimi gels in different treatment groups (A-C). Correlation analysis of lipid oxidation with odor compounds (D—H). Different uppercase letters indicated significant differences in the TBARS values of surimi gels under different frozen storage times (*p* < 0.05), and different lowercase letters indicated significant differences in the TBARS values of surimi gels with different cross-linking degrees (*p* < 0.05).

The hexanal content increased consistently with storage time, and compared to other groups, the LF-80 °C group showed a lower hexanal content. The increase in hexanal content after 90 days of storage was associated with intensified lipid oxidation in the surimi gels, with the highest hexanal content detected in the AF-18 °C group, which increased by 2.07 times (29.66 % cross-linking degree) and 2.00 times (62.99 % cross-linking degree) compared to the fresh control group (Fig. 2D), consistent with the changes in TBARS values indicating lipid oxidation (Fig. 3A-C). Furthermore, the contents of heptanal and benzaldehyde significantly increased (*p* < 0.05) with prolonged storage time (Fig. 2G-L). Heptanal, primarily derived from the oxidation of oleic and linoleic acids, possesses fatty, putrid, and pungent odors, and its increase during storage may negatively impact the flavor of surimi gels. These findings indicated that increasing the freezing rate and cross-linking degree was beneficial for reducing the accumulation of off-flavor compounds in surimi gels during storage.

Octanal content initially increased, peaking at 20 days of frozen storage before decreasing. The AF-18 °C group had the lowest octanal content at the end of storage (Fig. 2M). Similarly, nonanal showed a similar trend, reaching a maximum of 20 days. Compared to the fresh control, nonanal content increased by 48.22 %, 170.89 %, and 183.78 % at AF-18 °C, LF-35 °C, and LF-80 °C, respectively (29.66 % cross-linking degree); and by 44.17 %, 109.85 %, and 117.09 % (62.99 % cross-linking degree) (Fig. 2P-R). The AF-18 °C group had the lowest nonanal content at the end of storage, measuring 6.86 ± 1.39 μg/kg (29.66 % cross-linking) and 7.36 ± 1.03 μg/kg (62.99 % cross-linking), respectively. In contrast, the LF-80 °C group had the highest nonanal content, at 10.19 ± 2.66 μg/kg (29.66 % cross-linking) and 11.07 ± 0.04 μg/kg (62.99 % cross-linking), which were 44.08 % and 40.45 % lower than the peak levels at 20 days of storage, respectively. Increasing the cross-linking degree minimized the loss of nonanal after 90 days of storage in surimi gels. At the end of the storage period, the LF-80 °C group showed higher octanal and nonanal contents than the AF-18 °C and LF-35 °C groups, indicating that a higher freezing rate was more favorable for retaining beneficial volatile odor compounds during storage. In the early stages of storage, oxidation primarily affects the flavor of surimi gel. As storage time progresses, ice recrystallization enlarges crystals, damaging the gel structure and increasing moisture loss upon thawing. As a result, the samples' ability to retain flavor components is diminished, and more volatile compounds are released.

Both 1-octen-3-ol and 1-hexanol increased over storage time. The AF-18 °C group had the highest levels of these two compounds after 90 days, being 2.22 and 4.70 times (29.66 % cross-linking) and 1.77 and 3.24 times (62.99 % cross-linking) higher than the fresh control groups (Fig. 2S-X). Higher freezing rates and cross-linking reduced the accumulation of these compounds. Alcohols such as 1-octen-3-ol and 1-hexanol, generated from lipid oxidation, are associated with off-flavors including rancid and fishy odors (Zhou et al., 2024). 1-octen-3-ol, produced during lipid oxidation, contributes to meat spoilage and imparts a mushroom and fishy odor (Luo, Huang, Yu, et al., 2024). These results suggest that AF-18 °C treatment led to odor deterioration. In contrast, the high freezing rate in the LF-80 °C group resulted in the formation of smaller, uniform ice crystals, which minimized damage to the network structure, thereby better preserving the original flavor characteristics of the surimi gels at the end of storage. Additionally, by raising the cross-linking degree to 62.99 %, the surimi gel became denser and more homogeneous, and its rigidity increased, thereby enhancing its ability to resist ice crystal growth and reducing mechanical damage to the surimi gel. This damage reduction further reduced the loss of certain odor compounds during storage, thereby favorably maintaining the original flavor characteristics of the surimi gels.

### 3.4 Analysis of lipid oxidation and its correlation with odor compounds

The primary lipid oxidation products are malondialdehyde and various off-flavor carbonyl compounds, which can generate undesirable putrid odors, adversely affecting product quality. The TBARS (thiobarbituric acid reactive substances) values of surimi gels from different treatment groups significantly increased with storage time (*p* < 0.05) (Fig. 3A-C). The freezing method played a significant role in lipid oxidation during the storage of surimi gels with different cross-linking degrees. After 90 days of storage, the TBARS values of surimi gels frozen at AF-18 °C, LF-80 °C, and LF-35 °C reached their maximum values, increasing by 1.83, 1.50, and 1.36 times, respectively, compared to the fresh control group (29.66 % cross-linking degree), and by 1.69, 1.43, and 1.31 times for the 62.99 % cross-linking degree. This phenomenon may be attributed to the mechanical damage to the gel network caused by ice crystal formation and recrystallization, which promoted pro-oxidant release, thereby exacerbating lipid oxidation. Furthermore, under the expansion pressure caused by ice crystals, fatty acids in meat products are transported to the product surface, readily reacting with oxygen in the air, further facilitating lipid oxidation. Research indicated that the threshold level for TBARS spoilage is between 2 and 2.5 mg/kg (Wang et al., 2018). Lipid spoilage produces undesirable odors and acidity, consequently impacting the flavor quality of the product. The TBARS values of surimi gels from the LF-35 °C and LF-80 °C groups remained within the normal range throughout the entire storage period. In contrast, the TBARS values for the AF-18 °C group approached the spoilage threshold after 90 days of storage. Fat oxidation was also significantly associated with fishy compounds such as 1-hexanol, 1-octen-3-ol, and hexanal during the late frozen storage period (60–90 days) (Fig. 3G-H). This indicated that the freezing method plays a crucial role in the odor changes of surimi gels during long-term storage. Compared to air freezing, liquid nitrogen spray freezing increased the freezing rate, which could reduce lipid oxidation during storage. Moreover, lowering the liquid nitrogen spray freezing temperature to −80 °C effectively mitigated the degree of lipid oxidation, thereby better preserving the flavor quality of surimi gels during storage.

### 3.5 Lipidomic analysis of surimi gels across various treatment groups

#### 3.5.1 Analysis of lipid composition in surimi gels from different treatment groups

**Fig. 4:**
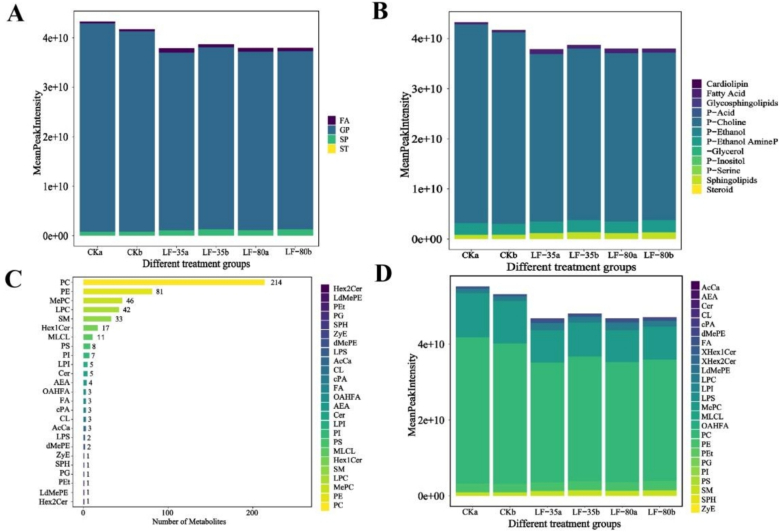
Lipid classification and content analysis of surimi gels in different treatment groups. CKa and CKb represented the surimi gels of the fresh control group with a degree of cross-linking of 29.66 % and 62.99 %, respectively; LF-35a and LF-35b represented the surimi gels with 29.66 % and 62.99 % cross-linking degree frozen by liquid nitrogen spray at −35 °C and frozen storage at −20 °C for 90 days, respectively; LF-80a and LF-80b represented the surimi gels with 29.66 % and 62.99 % cross-linking degrees respectively frozen by liquid nitrogen spray at −80 °C and frozen storage at −20 °C for 90 days.

A total of 498 lipid molecules were detected in the surimi gels from various treatment groups, which could be grouped into four classes (Fig. 4A): fatty acyls (FAs), glycerophospholipids (GPs), sphingolipids (SPs), and sterol lipids (STs). These classes further comprise 12 major categories (Fig. 4B) and 25 subclasses (Fig. 4C). As shown in Fig. 4A, glycerophospholipids (GPs) are the most abundant, followed by sphingolipids (SPs) and fatty acyls (FAs). Compared to the fresh control groups, the GPs content significantly decreased after frozen storage, with reductions of 14.52 % (LF-80a), 14.93 % (LF-35a), 11.22 % (LF-80b), and 9.33 % (LF-35b) observed (Fig. 4A). This indicated that increasing the cross-linking degree reduced the degradation of glycerophospholipid lipids of surimi gels during storage. Studies have shown that GPs are susceptible to degradation, leading to the formation of unsaturated fatty acids that impact product' flavor (Liu et al., 2023). Moreover, it was observed that phosphatidylcholine (PC), phosphatidylethanolamine (PE), methyl phosphatidylcholine (MePC), lysophosphatidylcholine (LPC), and sphingomyelin (SM) were the most prevalent lipid subclasses in the surimi gels, with quantities of 214, 81, 46, 42, and 33 species, respectively (Fig. 4C). Among all lipid subclasses, the PC peak signal intensity was the highest, indicating that PC was the dominant lipid subclass in the surimi gels, followed by MePC and PE (Fig. 4D). Compared to the CKa and CKb groups, the PC content in LF-80a, LF-35a, LF-80b, and LF-35b groups decreased by 17.96 %, 18.13 %, 13.70 %, and 11.45 %, respectively. It was noted that most of the reduced PC was composed of glycerolipids containing unsaturated fatty acids, such as docosapentaenoic acid (DPA, 22:5), docosahexaenoic acid (DHA, 22:6), and eicosapentaenoic acid (EPA, 20:5), specifically PC(20:5_22:6), PC(16:0_22:5), PC(20:4_22:6), PC(20:3_22:6), PC(16:0_20:5), and PC(18:0_20:5). Unsaturated fatty acids are susceptible to oxidative degradation during freezing. Given that PC contains various unsaturated fatty acids, it is inferred that the decrease in PC content after 90 days of storage was related to lipid oxidation.

The major fatty acids contained in PE are similar to those in PC. Studies have shown that the amino group on PE is more reactive than the double bond on fatty acyl residues, making it more likely to react with reactive oxygen species (ROS), free radicals, and oxidation products (Pongsetkul et al., 2017). After frozen storage, the signal intensity of most PE peaks, including PE(20:5_20:5), PE(18:3_20:5), PE(20:5_22:6), PE(20:5_18:2), PE(20:4_22:6), and PE(22:5_22:6), was lower than that of the fresh control groups. Research suggested that decreased PE and PC content was associated with lipid oxidation (Pei et al., 2025). This indicated that frozen storage altered the lipid distribution in surimi gels, consequently affecting their flavor quality. Additionally, MePC and phosphatidylglycerol (PG) also exhibited a decreasing trend after 90 days of storage. At 29.66 % cross-linking degree, MePC decreased by 27.78 % and 27.09 % in the LF-80a and LF-35a groups, respectively, compared to the CKa group, while PG decreased by 15.58 % and 22.46 %. When the cross-linking degree was raised to 62.99 %, MePC in the LF-80b and LF-35b treatment groups decreased by 22.28 % and 19.61 %, respectively, compared to the CKb group, while PG decreased by 2.93 % and 22.95 %. This suggested that MePC was more affected by the cross-linking degree, as increasing the cross-linking degree reduced the degradation of MePC. At the same time, PG was more influenced by the freezing method, where adopting −80 °C liquid nitrogen spray freezing decreased PG degradation during storage.

#### 3.5.2 Identification and analysis of differential lipids

**Fig. 5:**
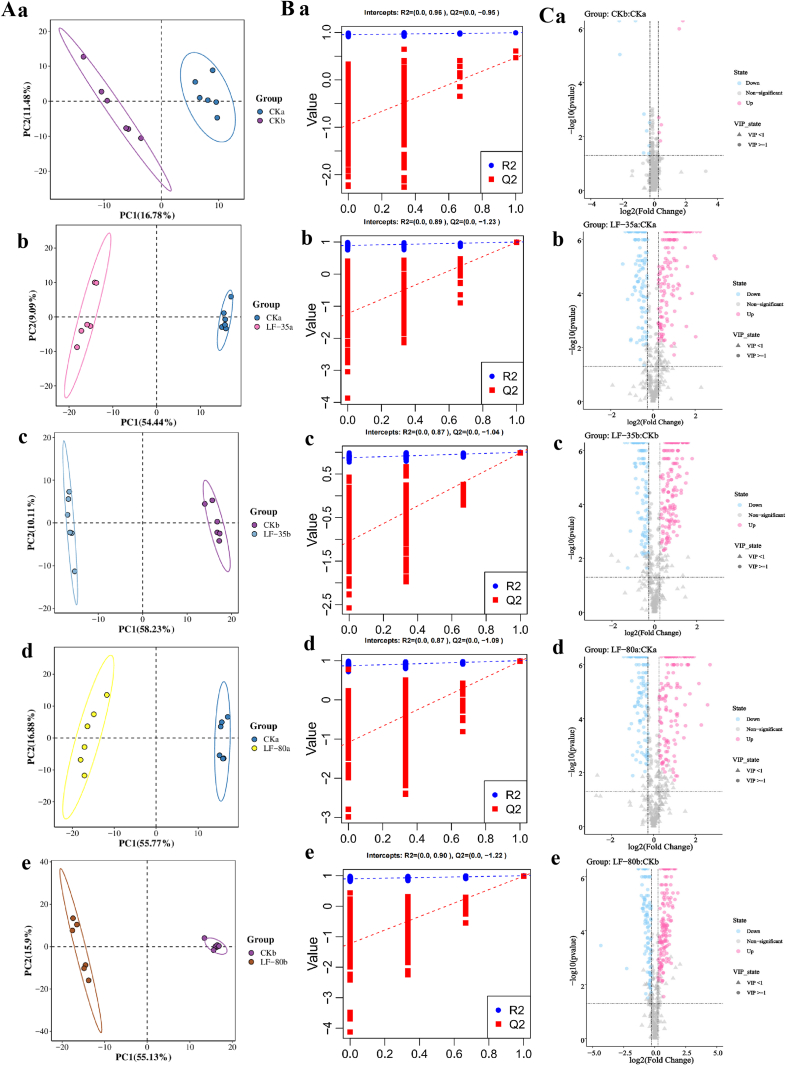
Analyze PLS-DA model score plots (A), permutation test plots (B), and volcano plot analysis (C) for differential lipids in surimi gels of different comparison groups. Each point in A represented a sample, different colors indicated different sample groups, and ellipses represented 95 % confidence intervals. The letters a,b,c,d, and e of B represented the CKb/CKa, LF-35a/CKa, LF-35b/CKb, and LF-80a/CKa, LF-80b/CKb comparison groups, respectively. Each point in C represented a differential lipid, where blue indicated down-regulated significant differential lipids, red indicated up-regulated significant differential lipids, and grey indicated non-significant differential lipids. Circles indicated differential lipids with VIP ≥ 1, and triangles were differential lipids with VIP < 1. (For interpretation of the references to color in this figure legend, the reader is referred to the web version of this article.)

The partial least squares discriminant analysis (PLS-DA) model was employed to investigate the overall variations in lipids among various comparison groups in the surimi gels. The results are displayed in Fig. 5A. To ensure the model's validity, the PLS-DA model underwent 200 permutation tests, and the intercept between Q^2^ and the vertical axis obtained from the general permutation test was less than 0, indicating that the model was reliable. The results are shown in Fig. 5 Ba-e. The PLS-DA model was valid and statistically significant, as all intercepts between Q^2^ and the vertical axis of the permutation test for the various comparison groups were less than 0.

As illustrated in Fig. 5A, the differential lipid profiles of surimi gels frozen at −35 °C and − 80 °C using liquid nitrogen and stored at −20 °C for 90 days significantly deviated from those of the fresh control group. Furthermore, the differential lipid profile of the surimi gels in the −35 °C liquid nitrogen freezing group was further away from that of the fresh control group compared to the −80 °C liquid nitrogen freezing group. This suggests that the freezing treatment has a significant impact on lipid changes in surimi gels, and the lipid alterations in low cross-linked surimi gels during storage are more influenced by the freezing method.

Applying the PLS-DA model with criteria of VIP ≥ 1, fold change (FC) > 1.2 or < 0.83, and *p* < 0.05, five comparative groups' (CKb/CKa, LF-80a/CKa, LF-35a/CKa, LF-80b/CKb, and LF-35b/CKb) differential lipid was identified and illustrated in Fig. 5C. The distribution of differential lipids in surimi gels from different comparison groups was visualized using a volcano plot. In the volcano plot, blue represented significantly downregulated differential lipids, while red indicated significantly upregulated differential lipids. The number of differential lipids in the fresh treatment groups of surimi gels with varying cross-linking degrees was relatively small (Fig. 5C). For instance, in the CKb/CKa comparison group, 15 significant differential lipids were identified, including 6 that were significantly upregulated, namely cyclic phosphatidic acid (cPA) (22:6), LPC (22:6), mono-lyso cardiolipin (MLCL) (58:14), lyso phosphatidylcholine (LPC) (20:4), LPC (18:2e), and lyso dimethyl phosphatidyl ethanolamine (LdMePE) (16:0). Conversely, 9 differential lipids were significantly downregulated, including fatty acid (FA) (20:5), LPC (18:0), LPC (20:1), N-acylethanolamine (AEA) (18:0), PC (32:3), PC (36:7e), PC (39:5e) (rep), PC (18:0_16:0), and PC (37:1).

The variety of differential lipids increased between the frozen groups and the fresh control groups. In the LF-35a/CKa comparison group, 251 differential lipids were identified, with 135 significantly upregulated differential lipids primarily consisting of 32 PC, 28 LPC, and 28 SM. There were also 116 significantly downregulated differential lipids, mainly composed of 75 PC, 19 MePC, and 15 PE. In the LF-35b/CKb comparison group, there were 254 differential lipids, including 145 significantly upregulated and 109 considerably downregulated. The upregulated differential lipids included 47 PC, 28 SM, 10 LPC, and other differential lipids, while the downregulated differential lipids included 59 PC, 25 PE, 14 MePC, and 11 other differential lipids. Research has indicated that lyso phospholipids are intermediates in phospholipid metabolism and can mediate phospholipid synthesis (Simons & Sampaio, 2011). LPC is a lyso phospholipid generated from phospholipid degradation (Cui et al., 2023). Therefore, it can be inferred that the significant upregulation of glycerophospholipids in surimi gels at the end of frozen storage may be related to phospholipid degradation (Liu et al., 2023).

Similarly, Jia et al. (2021) found that LPC significantly increased during low-temperature storage, and the lipid conversion between decreased PC and increased LPC was related to the biosynthetic pathways of glycerophospholipids (Zhang et al., 2023). In the LF-80a/CKa and LF-80b/CKb comparison groups, there were 247 and 255 differential lipids, respectively, with 128 and 153 significantly upregulated differential lipids, and 119 and 102 downregulated considerably differential lipids. Upregulated lipids mainly included SM, LPC, and PC, while downregulated ones were predominantly PC, MePC, and PE. PC and PE are the primary components of phospholipids in aquatic muscle tissue, playing a crucial role in regulating the physical structure and signal transduction of tissues (Fang et al., 2022). Reports indicated that polyunsaturated fatty acids, such as eicosapentaenoic acid (EPA) and docosahexaenoic acid (DHA), accumulate in the PE and PC components (Sun et al., 2021), and due to their high degree of unsaturation, PC and PE are prone to oxidation and hydrolysis. Compared to the fresh control group, the significant downregulation of most PC and PE in the surimi gels after 90 days of storage may have resulted in the severe structural damage caused by freezing, which induced the release of peroxidation free radicals, promoting the oxidation of polyunsaturated fatty acids and resulting in hydrogen rearrangement on the PC and PE chains and the cleavage of C—C groups of α-bonds (Liu et al., 2018)). MePC is formed through the methylation of PC; thus, when PC is downregulated, MePC also tends to show a downregulation trend. It has been reported that the oxidative degradation of polyunsaturated fatty acids is the leading cause of fishy odor (Li et al., 2022). Therefore, it can be inferred that the significant enhancement of fishy odor in surimi gels at the end of frozen storage was associated with the degradation of polyunsaturated fatty acids in PC and PE.

As demonstrated in Fig. S3, there were 103 common differential lipids presented in the four comparative groups (LF-80a/CKa, LF-80b/CKb, LF-35a/CKa, and LF-35b/CKb), containing 53 PC, 16 SM, 11 MePC, 10 PE, and others, indicating that low-temperature storage does not fully prevent lipid oxidation and degradation in surimi gels. The differences in lipids between the frozen surimi gels stored for 90 days and the fresh control group were primarily reflected in the PC, PE, SM, MePC, and LPC, which underwent significant changes during frozen storage and may serve as potential lipid biomarkers closely associated with the flavor quality changes in surimi gels. Research has indicated that the degradation of PC and PE can generate linoleic acid (18:2), α-linolenic acid, eicosatetraenoic acid, eicosapentaenoic acid, and docosahexaenoic acid, which are important precursors of flavor compounds (Zheng et al., 2022). After frozen storage, most PC and PE contents decreased, with a greater extent of change observed in the LF-35a group. Specifically, when the cross-linking degree was 29.66 %, compared to the fresh control group, unsaturated lipids such as PC (18:0_20:4), PE (20:5_20:5), and PE (18:3_20:5) decreased by 54.49 %, 77.08 %, and 69.83 %, respectively, under −35 °C liquid nitrogen spray freezing (Fig. S4). Increasing the freezing rate reduced the extent of the decline in these lipids, as under −80 °C liquid nitrogen spray freezing, the reductions were 53.10 %, 49.82 %, and 43.92 %, respectively. When the cross-linking degree was increased to 62.99 %, the reductions for these lipids were 49.59 %, 40.54 %, and 43.27 % (LF-35b), and 50.01 %, 38.71 %, and 40.11 % (LF-80b). These results indicated that both the freezing method and cross-linking degree significantly impacted lipid changes during the storage of surimi gels. Increasing the freezing rate and cross-linking degree mitigated the decline in specific differential lipids during the storage of surimi gels.

### 3.6 Correlation analysis of important volatile compounds with differential lipids

**Fig. 6:**
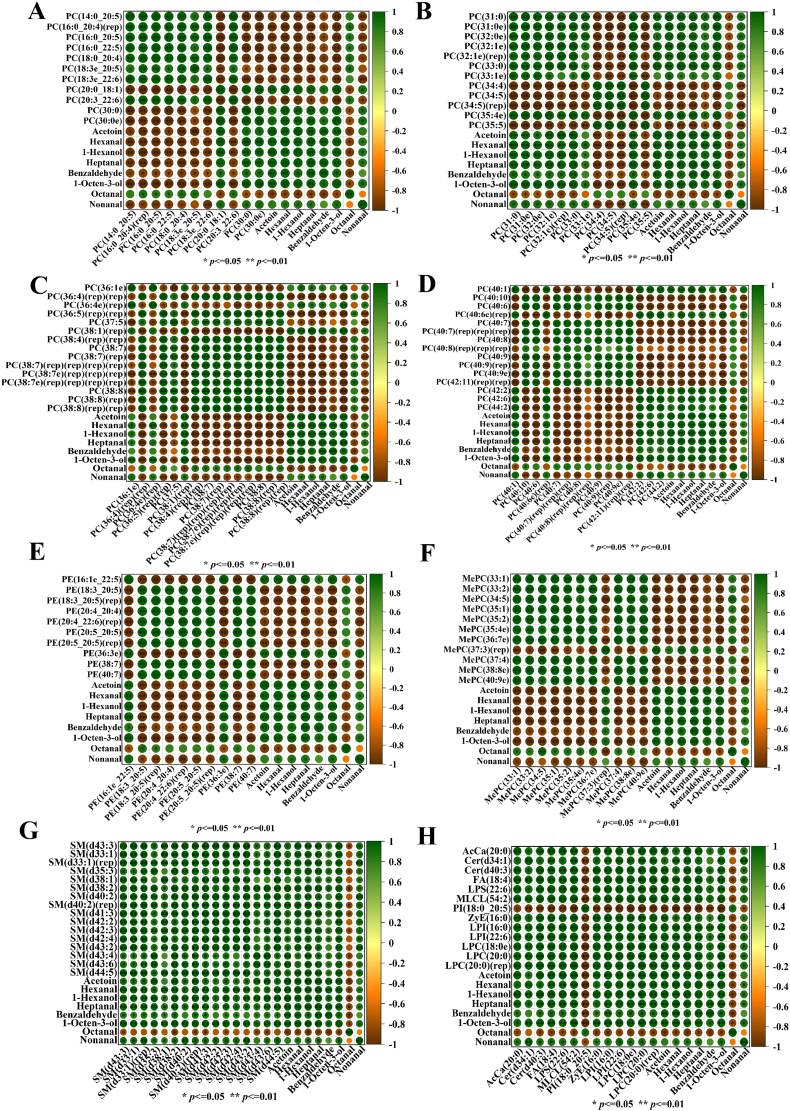
Correlation analysis of differential lipids with volatile odor compounds.

To reveal the effects of lipid degradation and oxidation on important volatile flavor compounds, a total of 103 different lipids and important volatile odor compounds shared between the LF-80a/CKa, LF-80b/CKb, LF-35a/CKa, and LF-35b/CKb comparison groups frozen at different freezing methods and frozen storage at −20 °C for 90 days as compared to the fresh control group were selected for correlation analysis, and the results were presented in Fig. 6. Most lipids showed a high correlation with volatile odor compounds (correlation coefficient R^2^ ≥ 0.90, *p* ≤ 0.05). Among these, 82 lipids were highly correlated with acetoin, while the numbers of lipids correlated with hexanal, heptanal, benzaldehyde, octanal, and nonanal were 96, 93, 22, 7, and 58, respectively. Lipids highly correlated with 1-octen-3-ol and 1-hexanol numbered 99 and 97, respectively. Most of these lipids were derived from PC and PE, with some originating from LPI, LPC, MePC, and SM. Among these, the unsaturated fatty acids in PC, including PC (14:0_20:5), PC (16:0_20:4)(rep), PC (16:0_20:5), PC (18:0_20:4), PC (18:3e_22:6), and PC (20:3_22:6), along with the unsaturated fatty acids in PE, such as PE (20:4_20:4) and PE (20:4_22:6)(rep), exhibited significant negative correlations with the volatile flavor compounds acetoin, hexanal, heptanal, benzaldehyde, nonanal, 1-octen-3-ol, and 1-hexanol (*p* ≤ 0.05).

Similarly, Zhang et al. (2024) showed that most PCs and PEs containing C18-C22 unsaturated fatty acids were negatively correlated with volatile compounds. This may be due to further oxidation of highly unstable primary oxidation products (hydroperoxides and conjugated dienes) produced by the reaction of unsaturated fatty acid double bonds in phospholipids with hydroxyl radicals to hydrocarbons such as alcohols, aldehydes and various flavor compounds (Mariutti & Bragagnolo, 2017). Previous studies have indicated that nonanal, hexanal, octanal, heptanal, and 1-octen-3-ol arise from the oxidative degradation of unsaturated fatty acids such as oleic acid, linoleic acid, α-linolenic acid, and arachidonic acid. Polyunsaturated fatty acids, including α-linolenic acid (18:3), arachidonic acid (20:4), EPA, and DHA, are distributed within PC and PE, which are classified as phospholipids. This suggested that phospholipids were crucial precursors of flavor compounds, and their degradation products significantly contributed to the flavor profile of the product. The results above indicated that the degradation of phospholipids during the frozen storage of surimi gels was a vital pathway for the formation of volatile odors. During storage, the degradation of phospholipids generates unsaturated fatty acids, which, upon further oxidative degradation, produce small molecular substances such as aldehydes, alcohols, and ketones, leading to the enhancement of product off-flavors (ichthyophilic, oily, and earthy flavors).

Additionally, It was also observed that LPI (16:0), LPI (22:6), LPC (18:0e), LPC (20:0), LPC (20:0)(rep), and various SMs, including SM(d43:3), SM(d33:1), SM(d33:1)(rep), SM(d35:3), SM(d38:1), SM(d38:2), SM(d40:2), SM(d40:2)(rep), SM(d41:3), SM(d42:2), SM(d42:3), SM(d42:4), SM(d43:2), SM(d43:4), SM(d43:6), and SM(d44:5), were all exhibited significant positive correlations (*p* ≤ 0.05) with acetoin, hexanal, heptanal, benzaldehyde, nonanal, 1-hexanol, and 1-octen-3-ol, while showing significant negative correlations with octanal (*p* ≤ 0.05). SM is produced through the transfer of phosphatidylcholine to Cer and consists of sphingosine, fatty acids, phosphate, and nitrogenous bases (Hannun & Obeid, 2018). Moreover, SM can degrade to yield various lipid-derived ceramides (Taniguchi & Okazaki, 2021). Studies indicated that the continuous conversion of SM to Cer may rapidly increase auto-oxidation, thereby deteriorating product quality (Jia et al., 2021).

## 4 Discussion

Lipidomics is an essential method for investigating the mechanisms of flavor formation induced by lipid oxidative degradation. It can be used to elucidate the production and reaction pathways of flavor precursors associated with lipids. To maintain the flavor quality of products during frozen storage and effectively prevent the accumulation of off-flavors, it is essential to investigate the mechanisms of flavor formation in aquatic products during frozen storage. Correlation analysis aids in understanding the relationships between variables. It plays a critical role in identifying precursor lipids and influencing factors involved in the formation of key volatile odor compounds during the frozen storage of surimi gels. The oxidative degradation of unsaturated fatty acids is a significant pathway for forming flavor compounds, as the oxidation of these acids generates various hydroperoxides. Hydroperoxides can originate from lipid auto-oxidation, where alkyl radicals generated in the initial stages of lipid molecular self-oxidation react with molecular oxygen to produce peroxy radicals, which then interact with hydrogen transferred from neighboring lipid molecules to form new alkyl radicals and hydrogen peroxide. Additionally, they can also derive from enzymatic oxidation, where unsaturated fatty acids are primarily oxidized to produce hydrogen peroxide through lipoxygenase dehydrogenation (Wang et al., 2025). Among them, volatile compounds derived from lipid autoxidation play a significant role in flavor formation.

Through lipidomics and correlation analysis, it was found that odor compounds were highly correlated with unsaturated fatty acids in PC, PE, LPI, LPC, MePC, and SM. Studies have suggested that phospholipids were the most susceptible lipids to changes during processing, and the unsaturated fatty acids produced from the degradation of phospholipids, such as linoleic acid, arachidonic acid, eicosatetraenoic acid, and alpha-linolenic acid, were important precursors for flavor formation (Liu, Lu, et al., 2024). The formation of linear aldehydes, such as nonanal, heptanal, and hexanal, in surimi gels during frozen storage is predominantly attributed to the peroxidation reactions of unsaturated fatty acids (Huang et al., 2025). Hexanal is mainly formed from the oxidation of linoleic acid and arachidonic acid (Zhou et al., 2024). Hexanal is known to be an aromatic active compound with a strong fatty odor, and high concentrations of hexanal are indicative of lipid oxidation, which is closely related to the generation of off-flavors (Yan et al., 2025). As the predominant volatile component, hexanal plays a key role in contributing to the fishy and rancid odor in aquatic products (Liu, Zhao, et al., 2024). Nonanal and octanal are primarily produced from the degradation of unsaturated fatty acids. Nonanal possesses floral and citrus aromas (Xu et al., 2023), and octanal exhibits fresh, grassy, and fatty odors (Li et al., 2022), both contribute positively to the flavor quality of surimi gels. Heptanal is produced through cleavage reactions during the oxidation degradation process of oleic acid, while diene aldehydes are formed through the self-oxidation of polyunsaturated fatty acids. Alcohols are major derivatives of lipid degradation, with 1-hexanol formed from the oxidation degradation of oleic acid and palmitic acid, while linoleic acid oxidation leads to the production of 1-octen-3-ol (Tu et al., 2025). Ketone compounds are typically produced from lipid oxidation, amino acid degradation, and the Maillard reaction contributing to fruity and creamy odors (Yang et al., 2025). The increase in acetoin content during storage may negatively affect the flavor of surimi gels, as it has a buttery, sweaty, and sour odor.

During frozen storage, the intensified lipid oxidation in surimi gels leads to the gradual accumulation of volatile odor substances such as hexanal, nonanal, benzaldehyde, 1-hexanol, and 1-octen-3-ol, which increased as the freezing rate decreased. The trends of hexanal, nonanal, and acetoin showed an initial increase followed by a decrease with increasing frozen storage time. This was related to the **worsening** of lipid oxidation due to freezing and may also be owing to the aggravated structural damage to the surimi gels network caused by ice crystal recrystallization during frozen storage, leading to the loss of some volatile odor compounds. The rapid freezing rate of LF-80 °C minimized structural disruption, while slower freezing in AF-18 °C exacerbated damage, potentially accelerating the generation of aldehydes, ketones, and esters that alter odor (Teng et al., 2023). Overall, combining LF-80 °C freezing with a higher cross-linking degree (62.99 %) was more effective in preserving the odor characteristics of surimi gels during frozen storage. Studies have reported that the production of fishy odors during the processing and storage of seafood products is associated with hexanal, heptanal, 1-octen-3-ol, dimethyl sulfide, and trimethylamine (Liu et al., 2024). The significant enhancement of fishy odors in surimi gels after frozen storage may be related to the accumulation of hexanal, heptanal, and 1-octen-3-ol. Research indicated that phospholipids are key precursors for most flavor compounds, and their degradation products significantly impact product flavor (Liu et al., 2023). The degradation of PC and PE generated linoleic acid, alpha-linolenic acid, and eicosatetraenoic acid. Unsaturated fatty acids such as oleic acid, linoleic acid, alpha-linolenic acid, and arachidonic acid are precursors for nonanal, hexanal, 1-octen-3-ol, heptanal, and hexanal. PC and PE are the main phospholipids in frozen surimi gels, and their degradation leads to the formation of linoleic acid, linolenic acid, and docosahexaenoic acid. Correlation analysis revealed that most unsaturated fatty acids in PC and PE were significantly correlated with volatile flavor compounds such as benzaldehyde, nonanal, 1-octen-3-ol, and 1-hexanol. Due to their high content of unsaturated fatty acids, PC and PE are susceptible to oxidative degradation. Compared with the fresh control group, the contents of most PC and PE in surimi gel significantly decreased after 90 days of frozen storage, accompanied by a marked increase in volatile compounds such as benzaldehyde, 1-octen-3-ol, and 1-hexanol. In summary, PC and PE were key lipids for flavor formation during the frozen storage of surimi gels with typical cross-linking degrees. The degradation of phospholipids and auto-oxidation of unsaturated fatty acids are important reaction pathways for forming volatile odor compounds.

## 5 Conclusion

The odor variations in surimi gels during frozen storage were primarily attributed to enhanced lipid oxidation and altered lipid distribution. A comparative analysis between frozen and fresh control groups (LF-80a/CKa, LF-80b/CKb, LF-35a/CKa, and LF-35b/CKb) identified 498 lipid molecules, among which 103 differential lipids were screened as potential markers related to odor changes. These differential lipids were mainly composed of PC, PE, LPI, LPC, MePC, and SM. After frozen storage, most PC, PE, and MePC lipids exhibited a decreasing trend, with oxidation and degradation of these lipids promoting the accumulation of volatile compounds such as hexanal, heptanal, and 1-octen-3-ol, thereby altering the odor profile of the surimi gel. This led to a significant decrease in fish aroma, while fishy, oil, and earthy odors intensified after 90 days of frozen storage. Increasing the freezing rate and cross-linking degree effectively reduced odor deterioration, as these factors slowed the oxidative degradation of certain unsaturated lipids, such as PC(18:0_20:4), PE(20:5_20:5), and PE(18:3_20:5), thus limiting the accumulation of off-odor compounds like hexanal, heptanal, 1-hexanol, and 1-octen-3-ol. Consequently, these strategies mitigated odor deterioration during frozen storage of surimi gels. The alterations in odor were primarily caused by lipid autoxidation and oxidative degradation of polyunsaturated fatty acids in phospholipids. This research offers valuable theoretical insights for enhancing the flavor quality of frozen surimi products.

## CRediT authorship contribution statement

**Xiaoying Luo:** Writing – original draft, Visualization, Validation, Software, Resources, Project administration, Methodology, Investigation, Funding acquisition, Formal analysis, Data curation, Conceptualization. **Guoyan Ren:** Writing – review & editing. **Shanbai Xiong:** Writing – review & editing, Supervision, Conceptualization. **Yueqi An:** Writing – review & editing. **Kang Huang:** Writing – review & editing. **Yang Hu:** Writing – review & editing, Supervision, Funding acquisition, Conceptualization.

## Declaration of competing interest

There are no conflicts of interest exists in the submission of this manuscript, and the manuscript is approved by all authors for publication.

## Data Availability

Data will be made available on request.

## Appendix A Supplementary data

Supplementary material Image 1
