# Supplementary material for: Mechanistic insights into volatile odor changes in surimi gels with typical cross-linking degrees during frozen storage based on lipidomics analysis
Source: Food Chem X. 2025 Jun 4;29:102630. doi: 10.1016/j.fochx.2025.102630 (PMC12178921; doi:10.1016/j.fochx.2025.102630)
Supplement: Supplementary file 1 — Supplementary material [file mmc1.docx]

*For submission to* ***Food Chemistry: X***

**Supplementary Data**

**Mechanistic insights into volatile odor changes in surimi gels with typical cross-linking degrees during frozen storage based on lipidomics analysis**

Xiaoying Luo^a^, Guoyan Ren^a^, Shanbai Xiong^b^, Yueqi An^c^, Kang Huang^d^, Yang Hu^b*^

^a^College of Food and Bioengineering, Henan University of Science and Technology, Luoyang, Henan, 471023, China

^b^College of Food Science and Technology, Huazhong Agricultural University, Wuhan, Hubei, 430070, China

^c^College of Health Science and Engineering, Hubei University, Wuhan, Hubei 430062, China

^d^Department of Biological Systems Engineering Washington State University Pullman, WA 99164, USA

**Correspondin*g author: E-mail, [huyang@mail.hzau.edu.cn](mailto:huyang@mail.hzau.edu.cn)

***
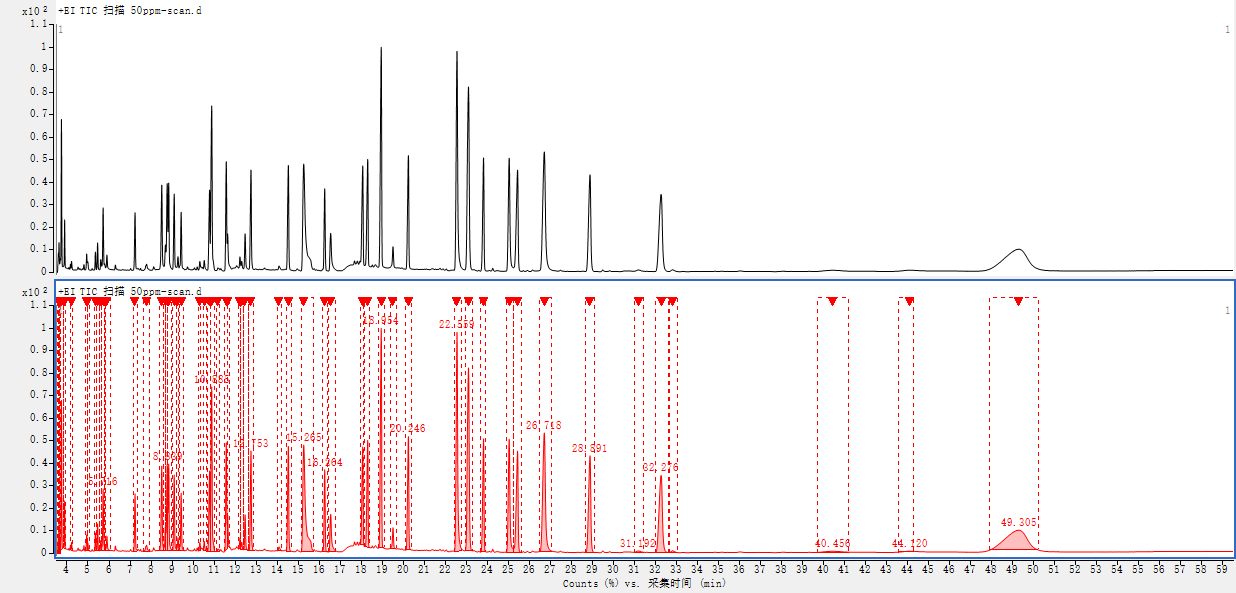
***

**Fig. S1** The chromatographic profiles of volatile odor compounds standards.

***
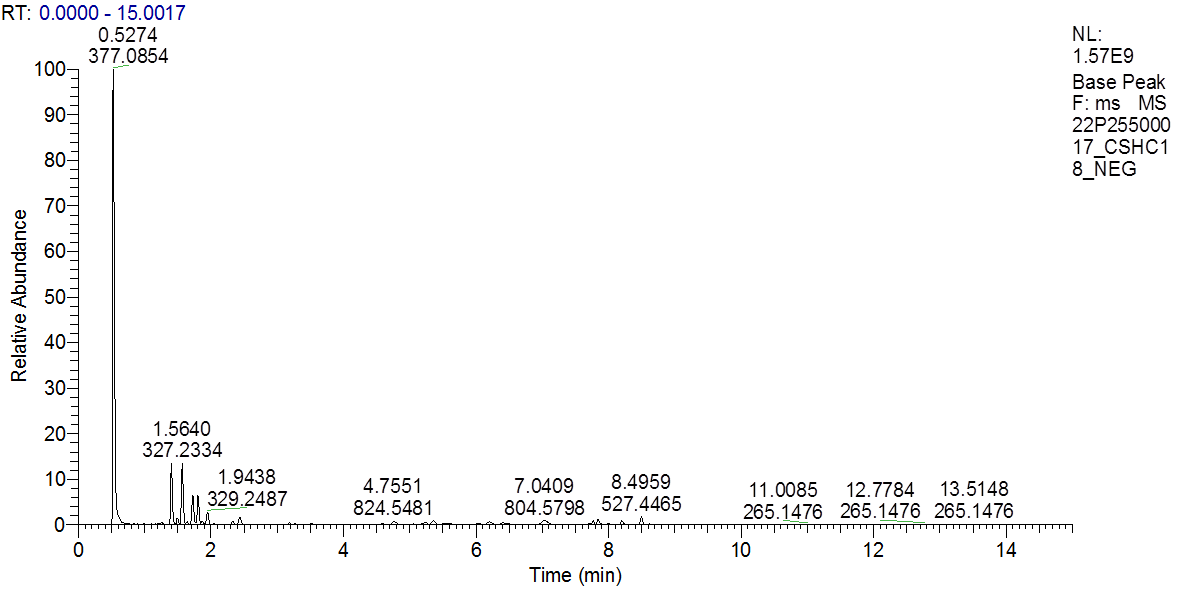
***

**Fig. S2** Representative base peak chromatograms of the samples in positive and negative ion modes.

**
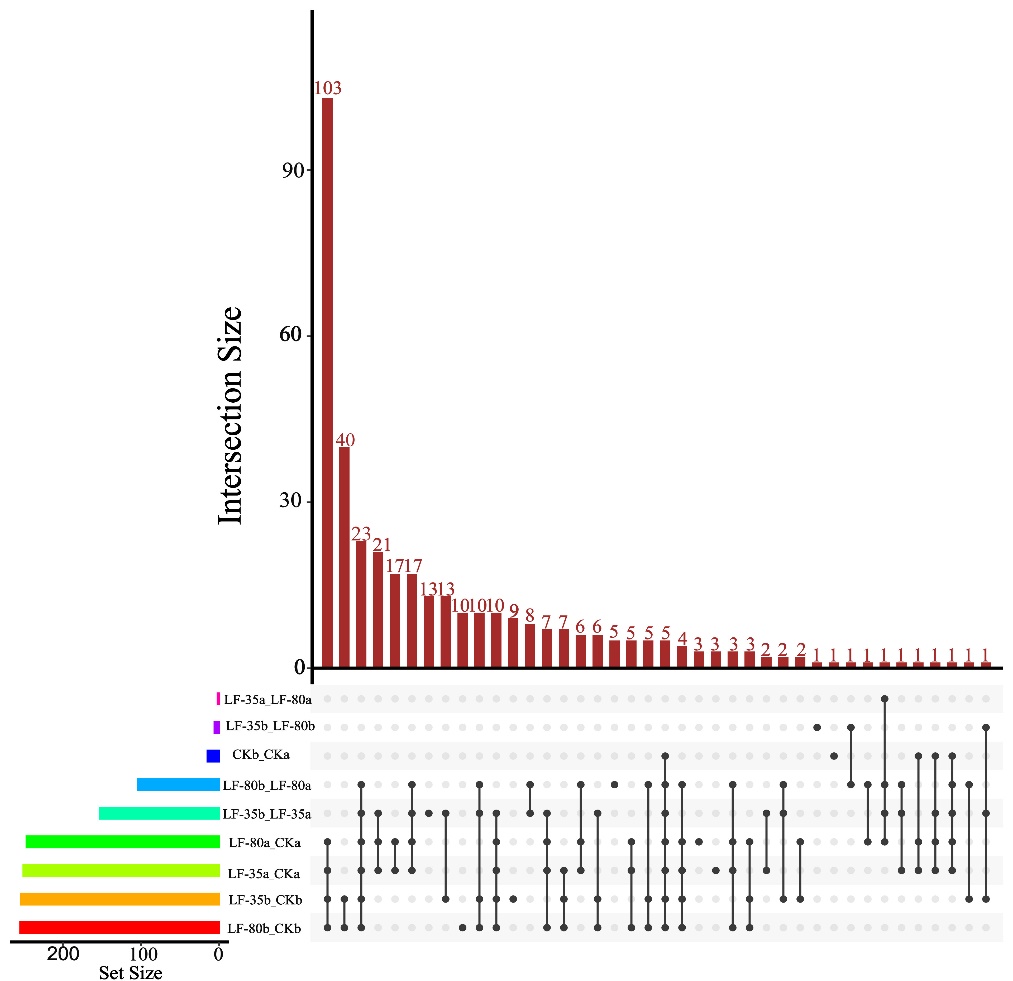
**

**Fig. S3** Upset plot analysis of differential lipids in different comparison groups.


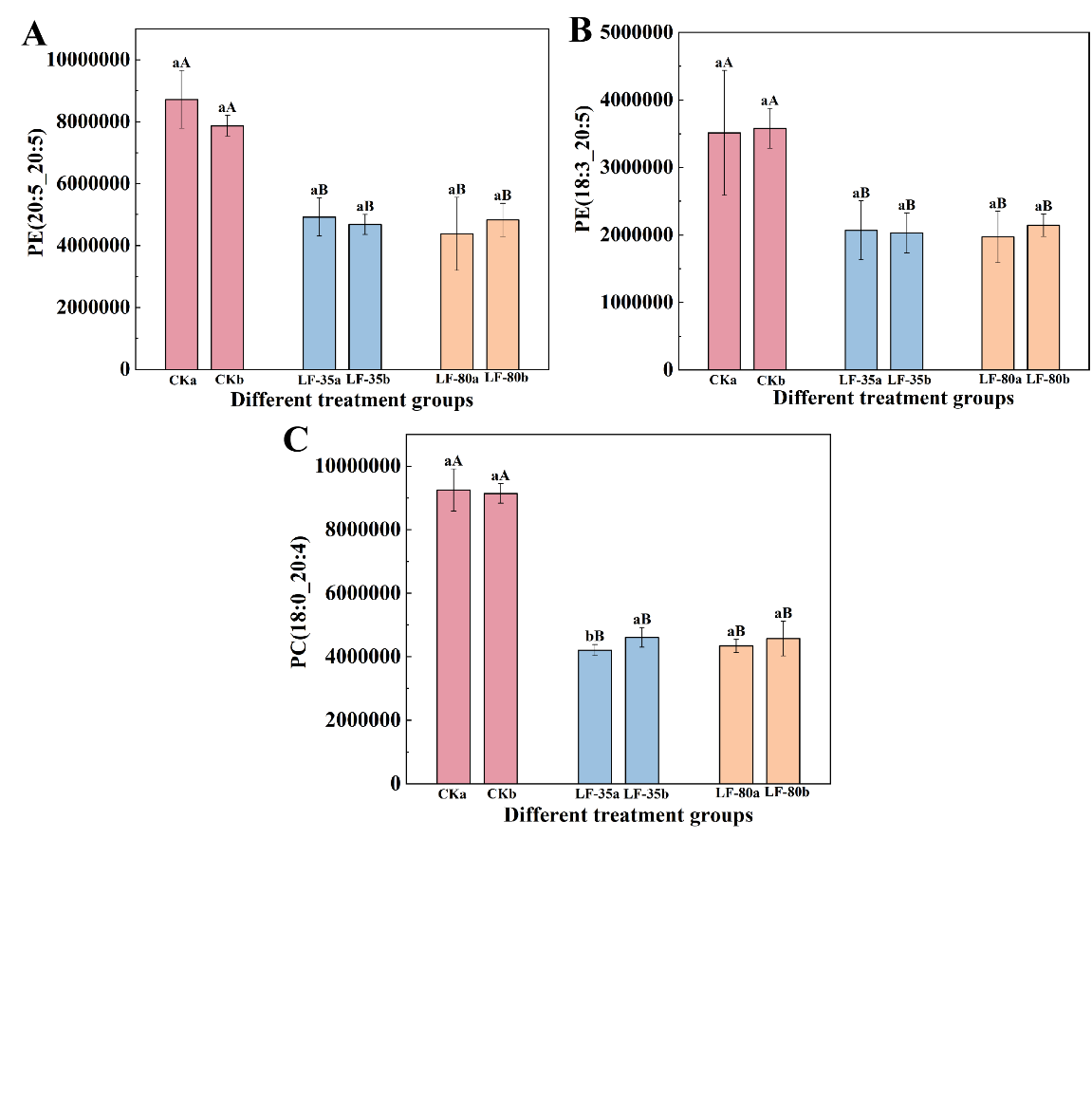


**Fig. S4** Analysis of differential lipids PE (20:5_20:5), PE (18:3_20:5), and PC (18:0_20:4).

| **Table S1** Standard curves of important volatile odor compounds in surimi gels | | |
| --- | --- | --- |
| Odor compounds | Standard curves | R^2^ |
| acetoin | y = 0.0063x + 0.026 | 0.9982 |
| hexanal | y = 0.028x - 0.0023 | 0.9999 |
| 1-hexanol | y = 0.0135x - 0.0587 | 0.9901 |
| heptanal | y = 0.0173x + 0.0193 | 0.9908 |
| benzaldehyde | y = 0.0428x + 0.0654 | 0.9884 |
| 1-octen-3-ol | y = 0.0142x + 0.0399 | 0.9945 |
| octanal | y = 0.0193x + 0.0403 | 0.9981 |
| nonanal | y = 0.0164x + 0.1553 | 0.9998 |
